# Stroke Aetiology and Collateral Status in Acute Ischemic Stroke Patients Receiving Reperfusion Therapy—A Meta-Analysis

**DOI:** 10.3390/neurolint13040060

**Published:** 2021-11-16

**Authors:** Akansha Sinha, Peter Stanwell, Roy G. Beran, Zeljka Calic, Murray C. Killingsworth, Sonu M. M. Bhaskar

**Affiliations:** 1 Neurovascular Imaging Laboratory, Clinical Sciences Stream, Ingham Institute for Applied Medical Research, Sydney, NSW 2170, Australia; akansha.sinha@student.unsw.edu.au (A.S.); roy.beran@unsw.edu.au (R.G.B.); Zeljka.Calic@health.nsw.gov.au (Z.C.); Murray.Killingsworth@health.nsw.gov.au (M.C.K.); 2 South-Western Sydney Clinical School, University of New South Wales (UNSW), Sydney, NSW 2170, Australia; 3 School of Health Sciences, University of Newcastle, Callaghan, Newcastle, NSW 2308, Australia; peter.stanwell@newcastle.edu.au; 4 NSW Brain Clot Bank, NSW Health Pathology, Sydney, NSW 2170, Australia; 5 Department of Neurology and Neurophysiology, Liverpool Hospital and South-Western Sydney Local Health District, Sydney, NSW 2170, Australia; 6 Medical School, Griffith University, Gold Coast, QLD 4222, Australia; 7 Faculty of Sociology, Sechenov Moscow First State University, 119991 Moscow, Russia; 8 Correlative Microscopy Facility, Department of Anatomical Pathology, NSW Health Pathology, Liverpool, NSW 2170, Australia

**Keywords:** collaterals, stroke, cerebrovascular disease, reperfusion therapy, aetiology, cardiovascular disease, neuroimaging

## Abstract

Background: The interplay between collateral status and stroke aetiology may be crucial in the evaluation and management of acute ischemic stroke (AIS). Our understanding of this relationship and its level of association remains sub-optimal. This study sought to examine the association of pre-intervention collateral status with stroke aetiology, specifically large artery atherosclerosis (LAA) and cardio-embolism (CE), in AIS patients receiving reperfusion therapy, by performing a meta-analysis. Methods: Relevant search terms were explored on Medline/PubMed, Embase and Cochrane databases. Studies were included using the following inclusion criteria: (a) patients aged 18 or above; (b) AIS patients; (c) patients receiving reperfusion therapy; (d) total cohort size of >20, and (e) qualitative or quantitative assessment of pre-intervention collateral status on imaging using a grading scale. Random-effects meta-analysis was performed to investigate the association of aetiology with pre-intervention collateral status, and forest plots of risk ratio (RR) were generated. Results: A meta-analysis was conducted on seven studies, with a cumulative cohort of 1235 patients, to assess the association of pre-intervention collateral status with stroke aetiology. Patients with LAA were associated significantly with an increased rate of good collaterals (RR 1.24; 95% CI 1.04–1.50; *p* = 0.020, z = 2.33). Contrarily, CE aetiology was associated significantly with a decreased rate of good collaterals (RR 0.83; 95% CI 0.71–0.98; *p* = 0.027, z = −2.213). Conclusions: This study demonstrates that, in AIS patients receiving reperfusion therapy, LAA and CE aetiologies are associated significantly with collateral status.

## 1. Introduction

Pre-intervention cerebral collateral status is an important consideration in the acute ischemic stroke (AIS) workup [1]. A crucial factor that impacts collateral recruitment is the underlying stroke aetiology [2]. AIS patients with underlying large artery atherosclerosis (LAA) aetiology have better pre-intervention collateral status—enhancing the potential to achieve improved clinical outcomes with reperfusion therapy [3,4]. In LAA, there is significantly increased shear pressure in cerebral vessels which promotes collateral formation [5]. Additionally, LAA is also linked to chronic cerebral hypoperfusion [2]. Chronic hypoperfusion may cause parenchyma to develop resilience to AIS. AIS with an embolic origin, such as cardio-embolic (CE) stroke secondary to atrial fibrillation, tends to cause sudden ischaemia. The delineation of the relationship of stroke aetiology with collateral status/recruitment is clinically relevant from a stroke diagnostic and prognostic standpoint and, hence, can assist in AIS clinical decision making. 

The current clinical assessment of stroke harnesses standardised methods, such as Trial of Org 10172 in Acute Stroke Treatment (TOAST), and Causative Classification of Stroke (CCS), to delineate the underlying aetiology [6]. These methods remain suboptimal and further avenues to refine aetiology assessment in stroke patients could be useful. This study sought to investigate the association of stroke aetiology, LAA vs. CE, with pre-intervention collateral status (good vs. poor) in AIS patients receiving reperfusion therapy (RT), specifically systemic thrombolysis (using tissue plasminogen activator (tPA)) and/or endovascular thrombectomy (EVT), by performing a meta-analysis.

The underlying question was, in AIS patients receiving RT:

Is stroke aetiology, LAA or CE, associated with pre-intervention collateral status?

## 2. Materials and Methods

### 2.1. Literature Search: Identification and Selection of Studies

**Figure 1:**
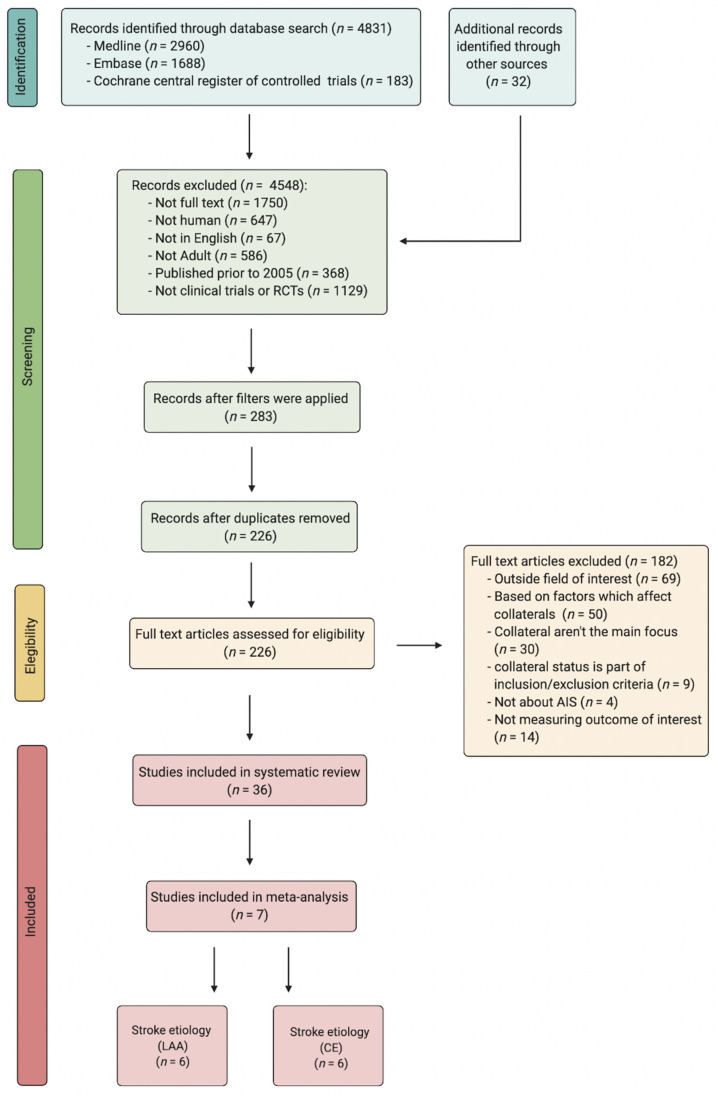
PRISMA flowchart detailing the process of finding and selecting studies for the meta-analysis. Abbreviations: RCT, randomised controlled trials; AIS, acute ischaemic stroke; LAA, large artery atherosclerosis; CE, cardio-embolism.

A Preferred Reporting Items for Systematic Reviews and Meta-Analyses (PRISMA) diagram explaining the search strategy, as well as details regarding the included studies, can be found in Figure 1. The protocol in this study adheres to the STARD-2015 guidelines (Supplemental Table S2), and the Meta-analysis Of Observational Studies in Epidemiology (MOOSE) checklist (Supplemental Table S3) [7]. Studies published in the English language investigating the impact of pre-intervention collateral status on AIS patients receiving RT with either LAA or CE as their stroke aetiology were reviewed on PubMed/Medline, Embase and the Cochrane Central Register of Controlled Trials for the period from January 2005 to June 2021. The search terms included: cerebral collateral, antegrade collateral, retrograde collateral and ischemic stroke, AIS, acute ischemic stroke anterior circulation, large vessel occlusion and reperfusion, endovascular treatment, tPA, EVT, clot retrieval, systemic thrombolysis and mechanical thrombectomy. The detailed search strategy can be found in the Supplementary Information (Search Terms). The following filters were applied: full text, English language, humans, and adults (>18 years) for the previously stated time frame.

### 2.2. Inclusion and Exclusion Criteria

The following inclusion criteria were applied: (a) patients aged > 18; (b) AIS patients; (c) patients receiving reperfusion therapy–either EVT and/or tPA; (d) total cohort size of >20, and (e) qualitative or quantitative assessment of pre-intervention collateral status on imaging using a grading scale. An outline of pre-intervention collateral grading scales, used by the included studies, is provided (Table 1). The following exclusion criteria were applied: (a) animal studies; (b) duplicated publications; (c) full-text article not available; (d) systematic review, meta-analysis, case conference summary; (e) texts in a language other than English and (f) data not stratified, according to the grading of pre-intervention collateral status (poor vs. good) and stroke aetiology (LAA vs. CE). 

### 2.3. Data Extraction

The titles and abstracts of studies selected from the literature search were screened for their eligibility in Endnote by two reviewers. All remaining articles were screened thoroughly to ensure they fitted within the eligibility criteria. The references of all included studies were screened for any additional studies that could be included. Any disagreement was resolved by consensus-based discussion. The following data were extracted from all included studies: (a) study details: author, title, year and country of publication; (b) patient demographics: cohort size in treatment (good collaterals) and control (poor collateral) groups, age and co-morbidities/risk factors; (c) pre-intervention collateral status (good or poor); (d) stroke aetiology of LAA or CE. All included studies dichotomised their patients into groups of good or poor collaterals based on their pre-intervention collateral status. Stroke aetiology was determined based on clinical assessment and/or the assessment of aetiology using TOAST or CCS classification. 

### 2.4. Quality Assessment of Included Studies 

The modified Jadad analysis, a scoring system that analyses the methodology of a trial, was used to assess the quality of each included study [13,14]. The risk of funding bias was also assessed by analysing the sources of funding for each study [15,16]. 

### 2.5. Statistical Analysis 

All statistical analyses were performed using STATA (version 13.0, StataCorp LLC, College Station, TX, USA). The pre-intervention characteristics of patients were recorded and converted from median and interquartile range (IQR) to mean and standard deviation (SD), where applicable [17]. The association of stroke aetiologies, specifically LAA and CE, with pre-intervention collateral status was investigated by performing a meta-analysis using DerSimonian and Laird random-effects modelling. Forest plots, containing summary effects for random-effects and inverse-variance weighted fixed-effect models, were generated to present the risk ratios (RR) (95% confidence intervals [CI]), percentage weights and the between-studies heterogeneity (I^2^ statistic, *p*-value). Additionally, summary effects and heterogeneity obtained from the meta-analysis (using the DerSimonian and Laird random-effects method, Mantel–Haenszel fixed-effect method and inverse-variance weighted fixed-effect) were also tabulated. An I^2^ of 75–100% is considerable, 50–90% is substantial, 30–60% is moderate and 0–40% is low heterogeneity, based on the Cochrane handbook [18]. The tests of overall effect drawn from the Z-test and *p*-values were also considered. The degree of inconsistency or heterogeneity across studies was quantified using the I^2^ index test and *p*-value. Other heterogeneity parameters including Cochran’s Q (heterogeneity in effect sizes), H (relative excess in Cochran’s Q over its degrees-of-freedom) and τ (heterogeneity variance estimate) test values obtained with the summary effects were also presented. Publication bias was assessed using funnel plots and Egger’s test of effect sizes. Using the “metainf” STATA command, analysis was also performed by excluding one study at a time to study the effect of one study on the overall effect. This helped assess the impact of each included study on the meta-analysis (Supplemental Figure S1). A *p*-value of <0.05 was considered statistically significant.

## 3. Results

### 3.1. Description of Included Studies 

A total of 7 studies were included in this study, with a cumulative cohort of 1235 patients [3,4,8,9,10,11,12]. Of these, 664 had good pre-intervention collateral status while 571 had poor pre-intervention collateral status. The mean age was 68.65 years (SD = 13.54). Pre-intervention systolic blood pressure was available for 419 patients, with a mean value of 143.6 (SD = 29.69). The clinical characteristics of all included studies, as well as the clinical outcomes they assess, can be found in Table 1. Collateral grading methods, as well as the description of the corresponding grading system/s, are provided in Table 1 and Supplemental Table S4. Data regarding risk factors and aetiology and the prevalence of each of them have been provided in Table 2. Effect size analysis for LAA or CE can be found in Supplemental Figure S2. The Jaded analysis and funding bias scores of each of the included studies can be found in Supplemental Table S1.

The summary effects and heterogeneity obtained from all included studies can be found in Table 3.

### 3.2. Association of Large Artery Atherosclerosis with Pre-Intervention Collateral Status

**Figure 2:**
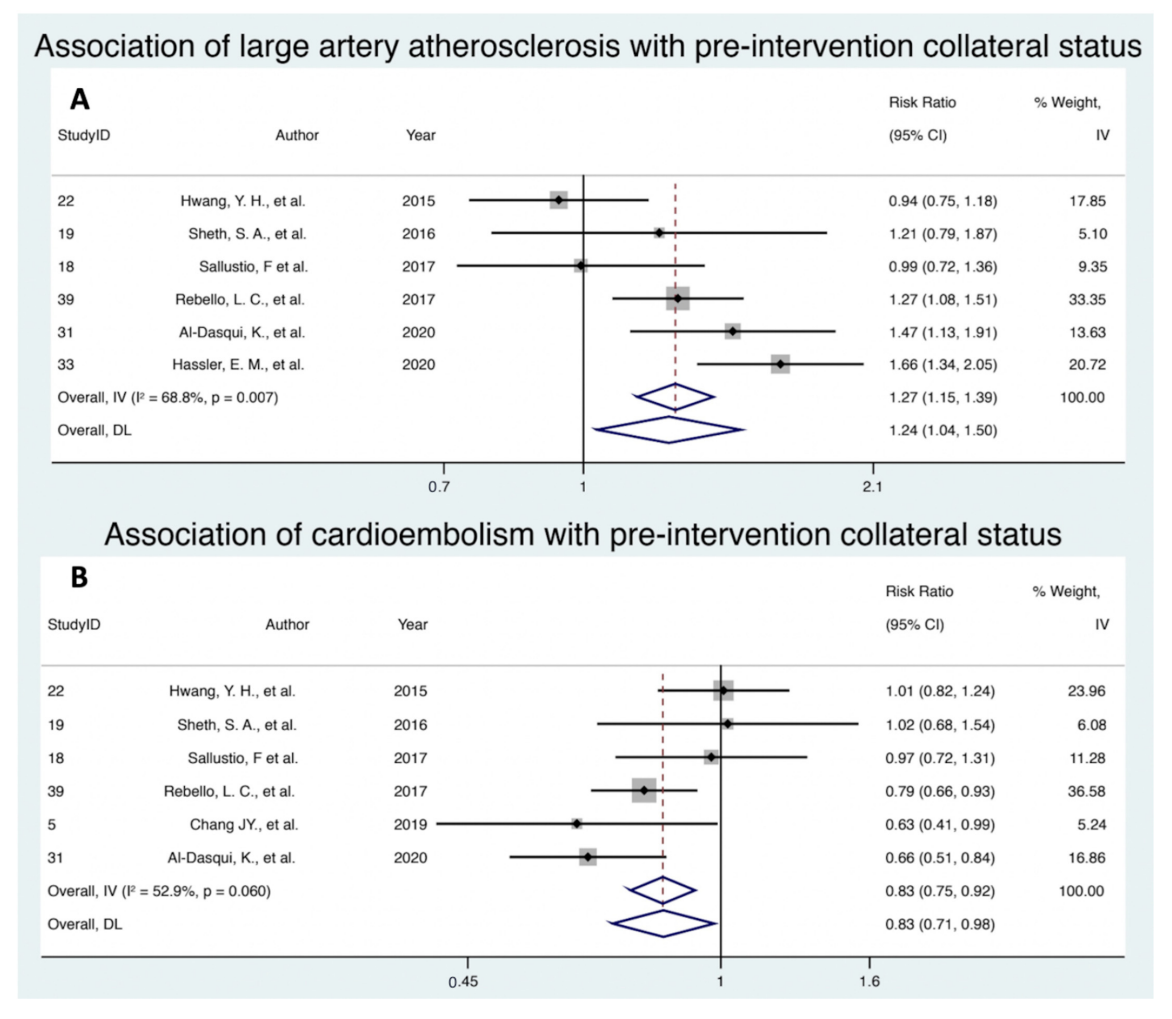
Forest plot showing the association of (**A**) large artery atherosclerosis stroke aetiology and (**B**) cardioembolism stroke aetiology with the pre-intervention collateral status in acute ischemic stroke patients receiving reperfusion therapy. Note: Random effect modelling (DL) values were used. Abbreviations: CI, confidence interval; IV, inverse variance; DL, DerSimonian and Laird.

Six studies assessed the association of LAA with pre-intervention collateral status, with a cumulative cohort of 1145 patients. Random effects modelling revealed that LAA was significantly associated with increased rates of good collaterals at pre-intervention (RR 1.24; 95% CI 1.04–1.50; *p* = 0.020, z = 2.33) (Figure 2A). There was substantial heterogeneity amongst the included studies (I^2^ = 68.8%; *p* = 0.007). Egger’s test and visual inspection of a funnel plot suggested the presence of some publication bias (e-value = 0.387) (Supplemental Figure S3A).

### 3.3. Association of Cardioembolism with Pre-Intervention Collateral Status

Six studies with a cumulative cohort of 954 patients investigated the association of CE and pre-intervention collateral status. Random effects modelling demonstrated that CE was associated significantly with increased rates of poor collaterals (RR 0.83; 95% CI 0.71–0.98; *p* = 0.027, z = −2.213) (Figure 2B). There was moderate to substantial heterogeneity amongst the included studies (I^2^ = 52.9%; *p* = 0.06). Egger’s test and visual inspection of a funnel plot suggested the presence of little to no publication bias (e-value = 0.629) (Supplemental Figure S3B).

## 4. Discussion

The results of this meta-analysis indicated that stroke aetiology was associated significantly with pre-intervention cerebral collateral status in AIS patients undergoing RT. Specifically, LAA was associated significantly with increased rates of good pre-intervention collaterals; whilst CE strokes were associated significantly with increased rates of poor pre-intervention collaterals. Collateral status in AIS is an important factor that has a role in mediating outcomes after RT [1,19]. Whilst previous meta-analyses have tried to analyse collateral status as a predictor of outcome in endovascular treatment of stroke [20,21]; to our knowledge, this is the first work to attempt to meta-analyse the association of collateral status with stroke aetiology.

The formation of cerebral collaterals can be affected by several environmental factors with the main factor in question relating to the presence of atherosclerotic plaques which obstruct cerebral blood flow. Plaques such as these alter haemodynamics within cerebral vessels, increasing shear pressure, thus activating endothelial cells and downstream signal transduction pathways, which contribute to the formation of collaterals and vascular remodelling [5]. This pathophysiological mechanism is responsible for the findings in a study by Rebello et al. wherein AIS patients with cervical atherosclerotic steno-occlusive disease had favourable pre-intervention collateral status when compared to those who experienced an embolic stroke, secondary to atrial fibrillation [4]. This association is also supported by Hassler et al. who noted that a pre-existing atherosclerotic extracranial ipsilateral carotid artery stenosis of ≥50% was associated with better collateral status [12]. This is consistent with the results of this meta-analysis wherein LAA was significantly associated with pre-intervention collateral status in AIS patients. 

Stroke aetiology may mediate collateral recruitment–potentially influencing response to time-critical reperfusion therapies in AIS [22]. This meta-analysis did not investigate this aspect. We postulate that in LAA patients, better collaterals develop over time in a proportion of patients resulting in high-grade stenosis [22]. Currently, data on whether stroke aetiology impacts reperfusion and outcomes after reperfusion therapy in AIS patients with large vessel occlusion in the anterior circulation, especially those treated with EVT or combined therapies (EVT ± IVT), are limited [23,24]. However, previous studies have shown that CE patients have worse outcomes than LAA patients [22,25,26], presumably due to greater successful reperfusion rates [25,26]. However, other studies found no statistically significant difference in successful reperfusion rates between LAA and CE, despite higher rates of favourable 3-month functional outcomes, post-reperfusion, for LAA [22,23,24]. Notably, in other studies, successful reperfusion is potentially more important for better outcomes and, specifically, more so in CE strokes than in the LAA [27]. It is worth noting that heart failure is more prevalent in stroke with CE than LAA, which may also contribute to poorer outcomes in the CE subgroup [28]. 

With regards to outcomes in AIS patients with CE aetiology, a recent study showed atrial fibrillation was associated with symptomatic intracerebral haemorrhage (sICH) in AIS patients treated with IVT [29]. This could be explained by the presence of poor collaterals in AF patients, or in AIS patients with CE aetiology, leading to an increased risk of sICH after reperfusion. A meta-analysis by Lu et al. about the safety and efficacy of IVT for AIS patients with AF and found worse outcomes in AIS patients with AF than those without AF. Authors also reported a higher incidence of sICH in AF patients than in non-AF patients (6.4% vs. 4.1%; *p*  <  0.001), as well as in AF patients receiving IVT compared to AF patients not receiving IVT (5.7% vs. 1.6%; *p*  <  0.001) [30].

As opposed to the chronic cerebral hypoperfusion induced collateral formation in LAA, the mechanism through which CE causes ischaemia is short-term and does not allow for collateral formation or vascular remodelling [5]. AIS patients with CE as their stroke aetiology are less likely to experience the benefits of good collateral supply. This is also seen in findings noted by Rebello et al., wherein AIS patients with underlying CE as their stroke aetiology do not associate with favourable pre-intervention collateral status [4]. Patients with CE are less likely to have good pre-intervention collateral status compared to those with LAA. It is worth noting that in stroke patients with intracranial atherosclerotic disease, concomitant systemic atherosclerosis (involving other arteries such as the extracranial carotid, coronary, aorta and lower extremity peripheral arteries) and overlapping stroke aetiologies, though less well studied [31], are not infrequent in clinical settings [32]. Hence, good collaterals may be observed in patients with embolic aetiology and co-existing LAA.

Another factor that has been shown to associate with pre-intervention collateral status is perilesional hyperperfusion (PLH). A prospective cohort found an independent association of PLH with good pre-intervention collateral status as well as major reperfusion at 24 h [19]. Pre-intervention collateral status was found to accurately predict PLH patterns, indicating an indirect role of PLH in prognosis [19]. The study also found that AIS patients with PLH were eight times more likely to experience HT when compared to patients without PLH [19]. The recruitment of immune cells following an ischemic event may be a contributing factor to this association [33]. This study used arterial spin labelling (ASL) to characterise PLH, demonstrating how advanced imaging, such as computed tomography perfusion (CTP) [34,35], CT angiography (CTA) [36] and ASL [19], have allowed quantitative estimation and characterisation of cerebral perfusion and the delineation of angiographic features including collateral status in AIS patients [14,16]. 

Multiphase CT angiography (mCTA) plays an important role in the localisation of occlusion as well as in the evaluation of spatial and temporal profile of the collateral status and its patency [1,37]. Rapid assessment of collateral circulation downstream of occlusion is of value in the selection of candidates for EVT [38]. Whilst qualitative scoring scales of collateral assessment are commonly used, they are limited due to complex method which may be time- and skill-intensive and their broader use is limited due to the lack of a standardised method [1,39]. Verdolotti et al. developed a simpler tool, Colorviz, which could be useful in the immediate evaluation of collaterals with comparable diagnostic evaluation to the mCTA. This could especially be useful for less experienced raters/clinicians [40].

Inflammatory cells play a crucial role in collaterogenesis, due to their ability to produce metalloproteinases and growth factors [5,41,42]. They are involved also in the formation of atherosclerotic plaques, thus highlighting their role in LAA strokes [43]. Semerano et al. found that lymphopenia and a high neutrophil-lymphocyte ratio (NLR), following an AIS, have been linked to poor clinical outcomes, especially in patients with good pre-intervention collateral status and successful reperfusion [33]. A higher neutrophil count one day after hospital admission was associated with sICH while a higher NLR was associated with parenchymal haemorrhage and sICH [33]. We postulate that the progression to poor outcomes despite good collateral status and successful reperfusion, e.g., in AIS patients with LAA, may be explained by other factors such as NLR [42] and severity of leukoaraiosis [44,45].

There are several limitations within the current study. A large majority of the included studies were retrospective, cross-sectional studies that provided a lower quality of evidence when compared to randomised clinical trials. However, since this current meta-analysis is not an investigation of outcomes, and since the specific research question of the association of stroke aetiology with collateral status is purely observational, it is not possible to answer this specific question. There were several limitations regarding the assessment of collateral status: single-phase computed tomography angiography is the most widely used imaging modality to assess collateral status. Due to its ability to visualise collaterals at a single point in time, it may not capture all collaterals that are present, thus underestimating the pre-intervention collateral status. The lack of a standardised grading system to assess collateral status is a source of heterogeneity that further impacts the reliability of the available data. Most of the included studies have used different grading methods (Table 1), which leads to inconsistencies in the objective definition of good versus poor collateral status. Further contributing to this limitation is the potential bias involved in the process of manually grading collaterals. Additionally, the different methods used to assess aetiology (such as TOAST or CCS) is another source of heterogeneity. Therefore, the findings of this meta-analysis should be interpreted in the context of study design and study population, limiting its generalisability to other study populations. The substantial heterogeneity amongst studies investigating the association of stroke aetiology with collateral status is also a limitation. Some studies included patients with a mixture of stroke aetiologies. However, given that groupwise data on collateral status were only available for CE and LAA aetiologies, the current study focused on these two specific aetiologies. Groupwise data on cryptogenic stroke and collateral status were not sufficient to merit inclusion in this current meta-analysis. Moreover, CE and LAA contribute to a majority of AIS patients in a real-world setting, therefore, this information can be of value in clinical practice. Moreover, we also acknowledge that some of these subgroups could have overlapping aetiologies, e.g., in Hassler et al. [12], 46 LAA subjects were only characterised by the presence of carotid artery stenosis but, among them, there were patients affected by atrial fibrillation, so they could be affected by cardioembolic strokes or, according to the TOAST classification, by strokes from an undetermined cause. Besides, given the varying pathogenesis of atherosclerotic occlusion based on the site of occlusion and heterogenous intervention protocols, it may be useful to compare LAA with CE for AIS with the same occlusion site [22]. 

Given that the random-effects model was used in the meta-analysis, some of these effects potentially would have been mitigated. There is a shortage of primary studies addressing the association between stroke aetiology and pre-intervention collateral status, thus limiting its evidence-based incorporation into clinical practice. Further high-quality studies are required to validate the findings of the present study. Future studies should aim to reduce heterogeneity associated with collateral grading methods and aetiology assessment tools as a means of improving the clinical applicability of their results. 

In conclusion, stroke aetiology is significantly associated with pre-intervention collateral status in AIS patients receiving RT. This meta-analysis also demonstrates that LAA is significantly associated with increased rates of good collaterals and CE with increased rates of poor collaterals. Despite limited primary studies, to the best of our knowledge, this is the first meta-analysis to investigate the association of stroke aetiology with pre-intervention collateral status. Gaining a better understanding of the association of stroke aetiology with pre-intervention collateral status may assist in the evaluation and management of AIS patients undergoing RT.

## Figures and Tables

**Table 1:** Clinical characteristics of studies included in the meta-analysis.

Study ID ^i^	Author	Year	Region	Study Type	Cohort	Reperfusion				Pre-Intervention Characteristics			Collateral Grading				Stroke Aetiology ^ii^					
						Reperfusion Modality	tPA *n* (%)	EVT *n* (%)	EVT ± tPA *n* (%)	Age, Years Mean ± SD	Sex, Male *n* (%)	Good Collaterals, *n* (%)	Imaging Modality	Collateral Grading Method	Definition of Good Collaterals ^iii^	Definition of Poor Collaterals ^iii^	LAA, *n* (%)			CE, *n* (%)		
																	O	GC	PC	O	GC	BC
5	Chang et al. [8]	2019	USA	Retrospective	90	EVT	NA	90 (100)	NA	72.3 ± 11.8	54 (60)	41 (45.6)	mCTA	Menon et al.	4 (when compared with the asymptomatic contralateral hemisphere, there is a delay of one phase in filling in of peripheral vessels, but prominence and extent is the same); 5 (there is no delay and normal or increased prominence of pial vessels/normal extent within the ischemic territory in the symptomatic hemisphere).	0 (when compared with the asymptomatic contralateral hemisphere, there are no vessels visible in any phase within the ischemic vascular territory); 1 (there are just a few vessels visible in any phase within the occluded vascular territory); 2 (there is a delay of two phases in filling in of peripheral vessels and decreased prominence and extent or a one-phase delay and some ischemic regions with no vessels); 3 (there is a delay of two phases in filling of peripheral vessels or there is a one-phase delay and significantly reduced number of vessels in the ischemic territory).	NA	NA	NA	54 (60)	20 (22.2)	34 (37.8)
18	Sallustio et al. [9]	2017	Italy	Retrospective	135	EVT ± tPA	79 (58.5)	135 (100)	79 (58.5)	68.3 ± 14.3	67 (49.6)	75 (55.6)	Cerebral angiography	Christoforidis et al.	1 (collaterals reconstituted the distal portion of the occluded vessel segment); 2 (collaterals reconstituted vessels in the proximal portion of the segment adjacent to the occluded vessel); 3 (collaterals reconstituted vessels in the distal portion of the segment adjacent to the occluded vessel); 4 (collaterals reconstituted vessels two segments distal to the occluded vessel); 5 (little or no significant reconstitution of the territory of the occluded vessel).		47 (34.8)	26 (19.3)	21 (15.6)	64 (47.4)	35 (26)	29 (21.5)
															“Good”	“Poor”						
19	Sheth et al. [10]	2016	USA	Retrospective	117	EVT ± tPA	59 (50.4)	96 (82.1)	NA ^iv^	66.7 ± 16.7	45 (38.5)	51 (43.6)	Cerebral angiography	ASITN/SIR	3 (collaterals with slow but complete angiographic blood flow of the ischemic bed by the late venous phase); 4 (complete and rapid collateral blood flow to the vascular bed in the entire ischemic territory by retrograde perfusion).	0 (no collaterals visible to the ischemic site); 1 (slow collaterals to the periphery of the ischemic site with the persistence of some of the defect); 2 (rapid collaterals to the periphery of ischemic site with the persistence of some of the defect and only a portion of the ischemic territory).	32 (27.3)	16 (13.6)	16 (13.7)	59 (50.4)	26 (22.2)	33 (28.2)
22 ^v^	Hwang et al. [11]	2015	Korea	Retrospective	207	EVT ± tPA ^vi^	103 (49.8)	NA ^vii^	NA ^vii^	67.1 ± 11.1	125 (60.4)	131 (63.3)	Cerebral angiography	ASITN/SIR	2 (Rapid collateral vessels to the periphery of ischemic site with the persistence of some of the defect and to only a portion of the ischemic territory); 3 (Collateral vessels with slow but complete angiographic blood flow of the ischemic bed by the late venous phase); 4 (Complete and rapid collateral blood flow to the vascular bed in the entire ischemic territory by retrograde perfusion).	0 (No collateral vessels visible to the ischemic site); 1 (Slow collateral vessels to the periphery of the ischemic site with the persistence of some of the defect).	66 (31.9)	40 (19.3)	26 (12.6)	107 (51.7)	68 (32.8)	39 (18.8)
31 ^viii^	Al-Dasqui et al. [3]	2020	USA	Retrospective	283	EVT ± tPA	130 (45.9)	270 (95.4)	NA ^ix^	69.2 ± 15.2	159 (56.2)	129 (45.6)	sCTA	Miteff	Good (major MCA branches reconstituted distal to the occlusion)	moderate (some MCA branches shown in the Sylvian fissure); poor (only distal superficial MCA branches reconstituted).	52 (18.4)	32 (11.3)	20 (7.1)	178 (62.9)	68 (24)	110 (38.9)
33	Hassler et al. [12]	2020	Austria	Retrospective	281	EVT ± tPA	166 (59.1)	281 (100)	166 (59.1)	68.6 ± 12.1	144 (51.2)	143 (50.9)	sCTA, MRI	Tan	2 (collateral supply filling >50% but <100%); 3 (100% collateral supply of the occluded MCA territory).	0 (absent collateral supply of the affected MCA territory); 1 (collateral supply filling ≤ 50%).	46 (16.4)	35 (12.5)	11 (3.9)	NA	NA	NA
39	Rebello et al. [4]	2017	USA	Retrospective	122	EVT ± tPA	54 (44.3)	122 (100)	54 (44.3)	69.7 ± 12.9	64 (52.5)	94 (77)	sCTA	Souza et al.	2 (diminished collaterals in <50% of the affected territory); 3 (collaterals equal to the contralateral side); 4 (increased collaterals).	0 (absent collaterals in >50% of the affected territory); 1 (diminished collaterals in >50% of the affected territory).	34 (27.9)	31 (25.4)	3 (2.5)	88 (72.1)	63 (51.6)	25 (20.5)

Abbreviations: O, overall; GC, good collaterals; PC, poor collaterals; NA, not applicable or the relevant information was not provided; USA, United States of America; tPA, tissue plasminogen activator; EVT, endovascular therapy; SD, standard deviation; LAA, large artery atherosclerosis; CE, cardioembolism. ^i^ Study ID is not consistently chronological; it is simply to assign all studies with an ID and does not hold any other significance. ^ii^ Data regarding stroke aetiology have been presented as overall values which have then been dichotomised into good collaterals and poor collaterals. ^iii^ Definition of good/poor pre-intervention collateral status used by the respective studies. ^iv^ In this study, all patients received either EVT (96 patients) or intra-arterial tPA (16 patients). Some, but not all, patients received intravenous tPA (43 patients). As such, 59 patients received tPA and 96 patients received EVT. The study does not disclose the number of patients who received a combination of EVT and tPA, although an obvious overlap can be seen in the number of patients who received EVT or tPA. ^v^ Note that this was the only study that provided data regarding the number of patients with stroke of undetermined aetiology/other determined aetiology, dichotomised into good and poor collaterals. A total of 34 patients had a stroke of undetermined aetiology/other determined aetiology, of which 23 had good collateral status while 11 had poor collateral status. ^vi^ This study does not specify whether all patients received EVT ± tPA or tPA ± EVT. As such, it was classified as EVT ± tPA, in line with all other studies included in this meta-analysis. ^vii^ This study acknowledges that “if treatable occlusion persisted, endovascular treatment was initiated”. The range of endovascular treatments includes “intra-arterial thrombolytic infusion (urokinase or rtPA), mechanical clot disruption, mechanical thrombectomy, rescue intra-/extra-cranial stent, or a combination”. Only the number of patients who received rtPA was specified, although it was acknowledged that some patients received a combination of treatments. ^viii^ A total of 53 patients had a stroke of undetermined aetiology. This was not dichotomised into good and poor collaterals. ^ix^ Similar to studyID 19, all patients received either EVT (270 patients) or intra-arterial tPA (17 patients). Some, but not all, patients received intravenous tPA (130 patients). As such, 147 patients received tPA and 130 patients received EVT. The study does not disclose the number of patients who received a combination of EVT and tPA, although an obvious overlap can be seen in the number of patients who received EVT or tPA.

**Table 2:** Overall summary of the prevalence of risk factors and stroke aetiologies in the meta-analysis.

Clinical Variable	Number of Patients with Data Available	Characteristics *n* (%)
Risk Factors		
Atrial fibrillation	952	466 (48.9)
Diabetes mellitus	952	194 (20.3)
Hyperlipidaemia	817	232 (28.4)
Hypertension	952	654 (67.7)
Coronary artery disease	297	44 (14.8)
Past stroke	414	80 (19.3)
Smoker	952	198 (20.8)
Aetiology		
Larger artery atherosclerosis	1145	277 (24.2)
Cardio embolism	954	550 (57.7)
Undetermined	490	87 (17.8)
Small vessel disease	117	1 (0.8)

**Table 3:** Summary effects and heterogeneity obtained from the meta-analysis of the association of pre-intervention collateral status with stroke aetiology.

Outcome	Effect Measure	Treatment Subgroup	Summary Effects						Heterogeneity ^¶^				Heterogeneity Variance Estimates
			REDL		FEMH		FEIV						
			RR (95% CI)	Tests of Overall Effect	RR (95% CI)	Tests of Overall Effect	RR (95% CI)	Tests of Overall Effect	Cochran’s Q	H	I^2^ *	*p*-Value	τ^2 †^
LAA	RR	EVT ± tPA	1.24 (1.04–1.50)	*p* = 0.02 z = 2.33	1.23 (1.11–1.36)	*p* < 0.0001 z = 3.87	1.27 (1.15–1.39)	*p* < 0.0001 z = 4.75	16.05	1.79	68.8	0.007	0.0346
CE	RR	EVT ± tPA	0.83 (0.71–0.98)	*p* = 0.027 z = −2.213	0.84 (0.75–0.94)	*p* = 0.002 z = −3.149	0.83 (0.75–0.92)	*p* < 0.0001 z = −3.526	10.61	1.46	52.9	0.060	0.0198

Abbreviations: LAA = Large Artery Atherosclerosis; CE = Cardioembolic; EVT = endovascular thrombectomy; tPA = transplasminogen activator; REDL = DerSimonian and Laird random-effects method; FEMH = Mantel–Haenszel fixed-effect method; FEIV = inverse-variance weighted fixed-effect; RR = Risk ratio; Q = Heterogeneity measures were calculated from the data with confidence intervals based on Cochran’s Q test; H = relative excess in Cochran’s Q over its degrees-of-freedom; I^2^ = proportion of total variation in effect estimate due to between-study heterogeneity (based on Cochran’s Q test); τ^2^ = among-study variance to test the comparisons of heterogeneity among subgroups; NA, not available/applicable. * Values of I^2^ are percentages. ^¶^ Heterogeneity measures were calculated from the data with 95% confidence intervals based on Gamma (random effects) distribution for Q. ^†^ Heterogeneity variance estimates (tau≤) were derived from the DerSimonian and Laird method.

## Data Availability

The original contributions presented in the study are included in the article/Supplementary Information, further inquiries can be directed to the corresponding author.

## Supplementary Materials

The following are available online at https://www.mdpi.com/article/10.3390/neurolint13040060/s1, Figure S1: Influence of a single study in meta-analysis estimation: (a) large artery atherosclerosis, (b) cardioembolism, Figure S2: Effect size analysis of all studies assessing the association between baseline collateral status and large artery atherosclerosis as an aetiology of stroke, Figure S3: Funnel plot displaying publication bias amongst all studies investigating the association of stroke aetiologies, (A) large artery atherosclerosis and (B) cardioemobolism, with pre-intervention collateral status, Table S1: Modified Jadad analysis scores and funding bias scores for each of the included studies, Table S2: STARD-2015 checklist, Table S3: MOOSE checklist for meta-analyses of observational Studies, Table S4: Description of the main baseline cerebral collaterals grading scales used by the included studies.
