# Supplementary material for: Stroke Aetiology and Collateral Status in Acute Ischemic Stroke Patients Receiving Reperfusion Therapy—A Meta-Analysis
Source: Neurol Int. 2021 Nov 16;13(4):608–21. doi: 10.3390/neurolint13040060 (PMC8628951; doi:10.3390/neurolint13040060)
Supplement: Supplementary file 1 [file neurolint-13-00060-s001.zip › neurolint-1421522-SI.pdf]

Supplementary Information

Stroke aetiology and collateral status in acute ischemic stroke patients receiving reperfusion therapy - a meta-analysis

List of Contents

1. **Search terms**
2. **Supplemental Tables**
   2a. **Supplemental Table S1.** Modified Jadad analysis scores and funding bias scores for each of the included studies.
   2b. **Supplemental Table S2.** STARD-2015 checklist
   2c. **Supplemental Table S3.** MOOSE checklist for meta-analyses of observational Studies
   2d. **Supplemental Table S4.** Description of the main baseline cerebral collaterals grading scales used by the included studies.
3. **Supplementary Figures**
   3a. **Supplemental Figure S1**: Influence of a single study in meta-analysis estimation: (a) large artery atherosclerosis, (b) cardioembolism
   3b. **Supplemental Figure S2**: Effect size analysis of all studies assessing the association between baseline collateral status and large artery atherosclerosis as an aetiology of stroke.
   3c. **Supplemental Figure S3.** Funnel plot displaying publication bias amongst all studies investigating the association of stroke aetiologies, (A) large artery atherosclerosis and (B) cardioemobolism, with pre-intervention collateral status
4. **List of References**

1. Search strategy

Collateral OR cerebral collateral OR pretreatment collateral OR collateral circulation OR antegrade collateral OR retrograde collateral OR collateral flow in the context of stroke AND stroke care OR acute stroke OR cerebrovascular accident OR brain ischemia OR ischemic stroke OR AIS OR acute ischemic stroke anterior circulation OR MCA stroke OR ICA stroke OR middle cerebral artery occlusion OR MCA occlusion OR ICA occlusion OR large vessel occlusion OR LVO OR cerebral infarction AND reperfusion OR endovascular thrombectomy OR endovascular treatment OR acute stroke treatment OR acute stroke intervention OR thrombolysis OR tPA OR thrombectomy OR EVT OR clot retrieval OR systemic thrombolysis OR mechanical thrombectomy OR endovascular procedure.

2. Supplemental tables

2a. Supplemental Table S1. Modified Jadad analysis scores and funding bias scores for each of the included studies.

Study ID	Author	MJA criteria								MJA score/8	MJA quality assessment	Funding bias score
		1	2	3	4	5	6	7	8			
5	Chang et al.	0	0	0	0	0	1	0	1	2	Low	0
18	Sallustio et al.	0	0	0.5	1	0	0	0	1	2.5	Low	1
19	Sheth et al.	0	0	0.5	1	0	0	0	1	2.5	Low	2
22	Hwang et al.	0	0	0.5	1	0	1	0	1	3.5	Low	0
31	Al-Dasqui et al.	0	0	0.5	1	0	1	0	1	3.5	Low	2
33	Hassler et al.	0	0	0.5	1	0	1	1	1	4.5	High	1
39	Rebello et al.	0	0	0.5	1	0	1	0	1	3.5	Low	0

Abbreviation: MJA, modified Jadad analysis.

Note: The numbers in the Modified Jadad Analysis criteria represent the following: **1**. Was the study randomised?; **2**. Was the method of randomisation appropriate?; **3**. Was the study described as being blinded?; **4.** Was the method of blinding appropriate?; **5**. Was there a description of withdrawals and dropouts?; **6**. Was there a clear description of the inclusion/exclusion criteria?; **7**. Was the method used to assess adverse events described?; **8**. Was the method of statistical analysis described?. The scores were assigned as follows: a score of 1 indicated “yes” and 0 indicated “no” or “not described”. A double blind was assigned a score of 1 while a single blind got 0.5. MJA quality assessment: low quality (0–3 points) and high quality (4–8 points) levels.

Funding bias was assessed using the following criteria: A high potential for bias – Score 3; If the study funded by industry – Score 2; Any conflict of interest declared relating to industry funding outside of the current research publications – Score 1; A study with low potential for bias – Score 0; A score of 1-2 indicated moderate potential for funding bi

2b. Supplemental Table S2. STARD-2015 checklist

Section & Topic	No	Item	Reported on page #
**TITLE OR ABSTRACT**			1
	**1**	Identification as a study of diagnostic accuracy using at least one measure of accuracy (such as sensitivity, specificity, predictive values, or AUC)	Not applicable
**ABSTRACT**			2
	**2**	Structured summary of study design, methods, results, and conclusions (for specific guidance, see STARD for Abstracts)	2
**INTRODUCTION**			3
	**3**	Scientific and clinical background, including the intended use and clinical role of the index test	3
	**4**	Study objectives and hypotheses	3
**METHODS**			4-6
Study design	**5**	Whether data collection was planned before the index test and reference standard were performed (prospective study) or after (retrospective study)	4-5
Participants	**6**	Eligibility criteria	4
	**7**	On what basis potentially eligible participants were identified<br>(such as symptoms, results from previous tests, inclusion in registry)	4
	**8**	Where and when potentially eligible participants were identified (setting, location, and dates)	Not applicable
	**9**	Whether participants formed a consecutive, random or convenience series	Not applicable
Test methods	**10a**	Index test, in sufficient detail to allow replication	4
	**10b**	Reference standard, in sufficient detail to allow replication	4
	**11**	Rationale for choosing the reference standard (if alternatives exist)	Not applicable
	**12a**	Definition of and rationale for test positivity cut-offs or result categories of the index test, distinguishing pre-specified from exploratory	4
	**12b**	Definition of and rationale for test positivity cut-offs or result categories of the reference standard, distinguishing pre-specified from exploratory	4
	**13a**	Whether clinical information and reference standard results were available to the performers/readers of the index test	Not applicable
	**13b**	Whether clinical information and index test results were available to the assessors of the reference standard	Not applicable

Analysis	**14**	Methods for estimating or comparing measures of diagnostic accuracy	Not applicable
	**15**	How indeterminate index test or reference standard results were handled	Not applicable
	**16**	How missing data on the index test and reference standard were handled	Not applicable
	**17**	Any analyses of variability in diagnostic accuracy, distinguishing pre-specified from exploratory	Not applicable
	**18**	Intended sample size and how it was determined	Not applicable
**RESULTS**			6-7, Figures 2-3
Participants	**19**	Flow of participants, using a diagram	Figure 1
	**20**	Baseline demographic and clinical characteristics of participants	6, Tables 1-2
	**21a**	Distribution of severity of disease in those with the target condition	6, Table 1
	**21b**	Distribution of alternative diagnoses in those without the target condition	Not applicable
	**22**	Time interval and any clinical interventions between index test and reference standard	Not applicable
Test results	**23**	Cross tabulation of the index test results (or their distribution)<br>by the results of the reference standard	Not applicable
	**24**	Estimates of diagnostic accuracy and their precision (such as 95% confidence intervals)	6-7, Tables 2-3
	**25**	Any adverse events from performing the index test or the reference standard	Not applicable
**DISCUSSION**			7-10
	**26**	Study limitations, including sources of potential bias, statistical uncertainty, and generalisability	8-10
	**27**	Implications for practice, including the intended use and clinical role of the index test	9-10
**OTHER INFORMATION**			
	**28**	Registration number and name of registry	Not applicable
	**29**	Where the full study protocol can be accessed	Not applicable
	**30**	Sources of funding and other support; role of funders	Not applicable

2c. Supplemental Table S3. MOOSE checklist for meta-analyses of observational Studies

Item No	Recommendation	Reported on Page No
Reporting of background should include		
1	Problem definition	3
2	Hypothesis statement	3
3	Description of study outcome(s)	3
4	Type of exposure or intervention used	4-5
5	Type of study designs used	Table 1, Figure 1
6	Study population	6, Table 1
Reporting of search strategy should include		
7	Qualifications of searchers (e.g., librarians and investigators)	1
8	Search strategy, including time-period included in the synthesis and key words	4, Supplementary Information
9	Effort to include all available studies, including contact with authors	4
10	Databases and registries searched	4
11	Search software used, name and version, including special features used (eg, explosion)	4
12	Use of hand searching (eg, reference lists of obtained articles)	4-5
13	List of citations located and those excluded, including justification	6, Figure 1
14	Method of addressing articles published in languages other than English	4
15	Method of handling abstracts and unpublished studies	4
16	Description of any contact with authors	NA
Reporting of methods should include		

17	Description of relevance or appropriateness of studies assembled for assessing the hypothesis to be tested	Supplementary Information
18	Rationale for the selection and coding of data (eg, sound clinical principles or convenience)	4-5
19	Documentation of how data were classified and coded (eg, multiple raters, blinding and interrater reliability)	4-5
20	Assessment of confounding (eg., comparability of cases and controls in studies where appropriate)	NA
21	Assessment of study quality, including blinding of quality assessors, stratification, or regression on possible predictors of study results	5, Supplementary Information
22	Assessment of heterogeneity	5-6
23	Description of statistical methods (eg., complete description of fixed or random effects models, justification of whether the chosen models account for predictors of study results, dose-response models, or cumulative meta-analysis) in sufficient detail to be replicated	5-6
24	Provision of appropriate tables and graphics	Tables 1-3, Figures 1-3
Reporting of results should include		
25	Graphic summarizing individual study estimates and overall estimate	Figures 2-3
26	Table giving descriptive information for each study included	Table 1
27	Results of sensitivity testing (eg, subgroup analysis)	Figures 2-3
28	Indication of statistical uncertainty of findings	6-7, Figures 2-3

Reporting of discussion should include		
29	Quantitative assessment of bias (eg., publication bias)	Supplementary Information
30	Justification for exclusion (eg., exclusion of non-English language citations)	4, Figure 1
31	Assessment of quality of included studies	Supplementary Information
Reporting of conclusions should include		

32	Consideration of alternative explanations for observed results	7-10
33	Generalization of the conclusions (i.e., appropriate for the data presented and within the domain of the literature review)	8-9
34	Guidelines for future research	9-10
35	Disclosure of funding source	10

From: Stroup DF, Berlin JA, Morton SC, et al, for the Meta-analysis Of Observational Studies in Epidemiology (MOOSE) Group. Meta-analysis of Observational Studies in Epidemiology. A Proposal for Reporting. *JAMA*. 2000;283(15):2008-2012. doi: 10.1001/jama.283.15.2008.

2d. Supplemental Table S4. Description of the main baseline cerebral collaterals grading scales used by the included studies.

Grading scale	Description
Tan (Tan *et al.*, 2009)	0: absent collateral supply to the occluded MCA territory<br>1: collateral supply filling ≤50% but >0% of the occluded MCA territory<br>2: collateral supply filling >50% but <100% of the occluded MCA territory<br>3: 100% collateral supply of the occluded MCA territory
Maas (Maas *et al.*, 2009)	1: no vessel opacification<br>2: less than those on the contralateral side<br>3: equal to those on the contralateral side<br>4: more than those on the contralateral side<br>5: exuberant
Miteff (Miteff *et al.*, 2009)	1: contrast opacification is seen only in the distal superficial branches<br>2: some vessels are seen at Sylvian fissure<br>3: vessels are reconstituted distal to the occlusion
ASITN/SIR (Zaidat *et al.*, 2013)	0: no collateral vessels visible to the ischaemic site<br>1: slow collateral vessels to the periphery of the ischaemic site with persistence of some of the defect<br>2: rapid collateral vessels to the periphery of the ischaemic site with persistence of some of the defect and to only a portion of the ischaemic territory<br>3: collateral vessels with slow but complete angiographic blood flow of the ischaemic bed by the late venous phase<br>4: complete and rapid collateral blood flow to the vascular bed in the entire ischaemic territory by retrograde perfusion.

Abbreviations: MCA, middle cerebral artery; ASITN/SIR, American Society of Intervention and Therapeutic Neuroradiology/Society of Interventional Radiology.

3. Supplementary Figures

3a. Supplemental Figure S1: **Influence of a single study in meta-analysis estimation for: (a) large artery atherosclerosis, (b) cardioembolism**

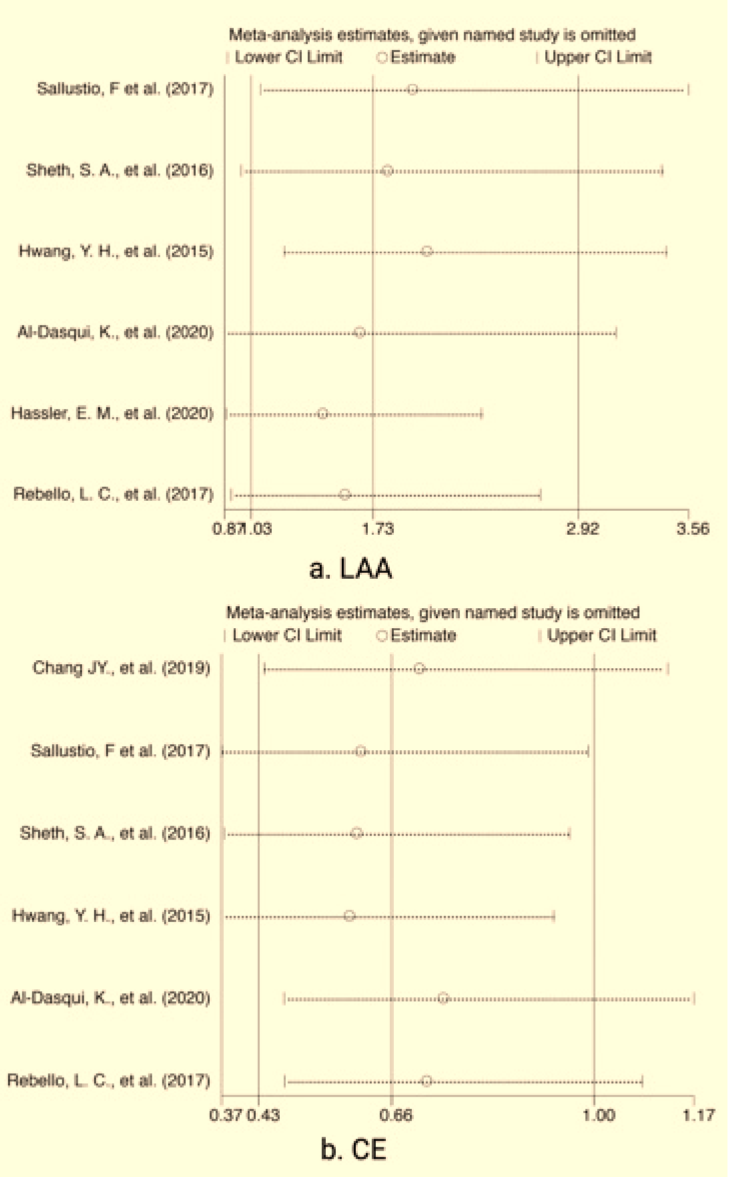

Abbreviations: LAA, large artery atherosclerosis; CE, cardioembolism.

3b. Supplemental Figure S2. Effect size analysis of all studies assessing the association between baseline collateral status and large artery atherosclerosis as an aetiology of stroke.

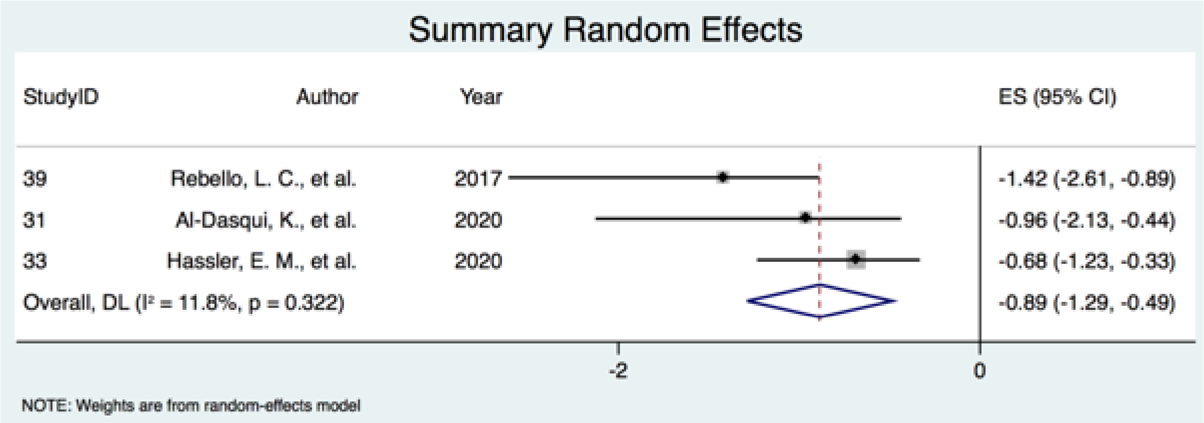

Note: Effect size could not be analysed for cardioembolism stroke aetiology.
Abbreviations: CI, confidence interval; ES, effect size

3c. **Supplemental Figure S3. Funnel plot displaying publication bias amongst all studies investigating the association of stroke aetiologies, (A) large artery atherosclerosis and (B) cardioemobolism, with pre-intervention collateral status**

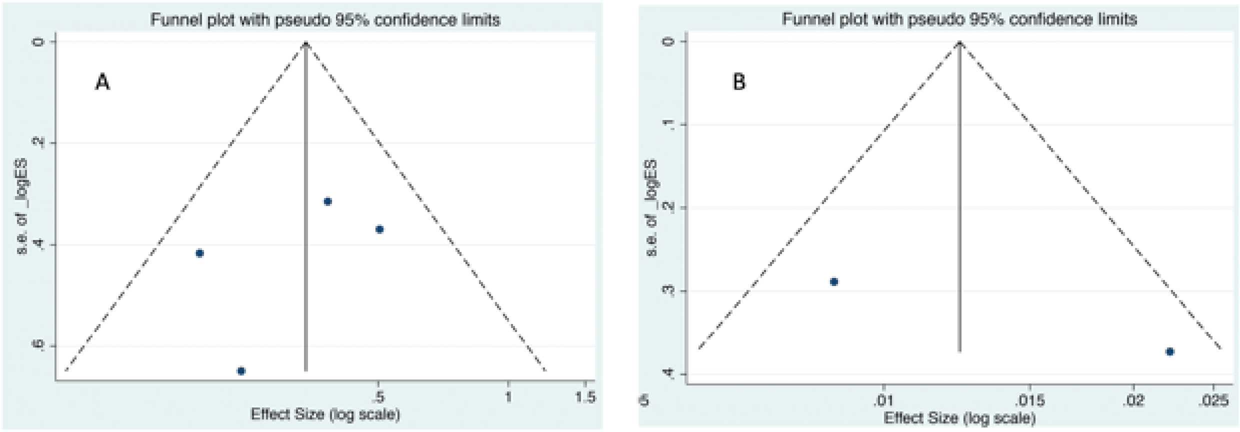

4. List of References

Al-Dasuqi, K., Payabvash, S., Torres-Flores, G.A., Strander, S.M., Nguyen, C.K., Peshwe, K.U., Kodali, S., Silverman, A., Malhotra, A., Johnson, M.H., Matouk, C.C., Schindler, J.L., Sansing, L.H., Falcone, G.J., Sheth, K.N. & Petersen, N.H. (2020) Effects of Collateral Status on Infarct Distribution Following Endovascular Therapy in Large Vessel Occlusion Stroke. *Stroke*, **51**, e193-e202.

Chang, J.Y., Jeon, S.B., Jung, C., Gwak, D.S. & Han, M.K. (2019) Postreperfusion Blood Pressure Variability After Endovascular Thrombectomy Affects Outcomes in Acute Ischemic Stroke Patients With Poor Collateral Circulation. *Front Neurol*, **10**, 346.

Hassler, E., Kneihsl, M., Deutschmann, H., Hinteregger, N., Magyar, M., Wießpeiner, U., Haidegger, M., Fandler-Höfler, S., Eppinger, S., Niederkorn, K., Enzinger, C., Fazekas, F. & Gattringer, T. (2020) Relationship between stroke etiology and collateral status in anterior circulation large vessel occlusion. *Journal of Neurology*, **267**, 3362-3370.

Hwang, Y.H., Kang, D.H., Kim, Y.W., Kim, Y.S., Park, S.P. & Liebeskind, D.S. (2015) Impact of time-to-reperfusion on outcome in patients with poor collaterals. *AJNR Am J Neuroradiol*, **36**, 495-500.

Maas, M.B., Lev, M.H., Ay, H., Singhal, A.B., Greer, D.M., Smith, W.S., Harris, G.J., Halpern, E., Kemmling, A., Koroshetz, W.J. & Furie, K.L. (2009) Collateral Vessels on CT Angiography Predict Outcome in Acute Ischemic Stroke. *Stroke*, **40**, 3001-3005.

Miteff, F., Levi, C.R., Bateman, G.A., Spratt, N., McElduff, P. & Parsons, M.W. (2009) The independent predictive utility of computed tomography angiographic collateral status in acute ischaemic stroke. *Brain*, **132**, 2231-2238.

Rebello, L.C., Bouslama, M., Haussen, D.C., Grossberg, J.A., Dehkharghani, S., Anderson, A., Belagaje, S.R., Bianchi, N.A., Grigoryan, M., Frankel, M.R. & Nogueira, R.G. (2017) Stroke etiology and collaterals: atheroembolic strokes have greater collateral recruitment than cardioembolic strokes. *Eur J Neurol*, **24**, 762-767.

Sallustio, F., Motta, C., Pizzuto, S., Diomedi, M., Giordano, A., D'Agostino, V.C., Samà, D., Mangiafico, S., Saia, V., Legramante, J.M., Konda, D., Pampana, E., Floris, R., Stanzione, P., Gandini, R. & Koch, G. (2017) CT angiography-based collateral flow and time to reperfusion are strong predictors of outcome in endovascular treatment of patients with stroke. *J Neurointerv Surg*, **9**, 940-943.

Sheth, S.A., Sanossian, N., Hao, Q., Starkman, S., Ali, L.K., Kim, D., Gonzalez, N.R., Tateshima, S., Jahan, R., Duckwiler, G.R., Saver, J.L., Vinuela, F. & Liebeskind, D.S. (2016) Collateral flow as causative of good outcomes in endovascular stroke therapy. *J Neurointerv Surg*, **8**, 2-7.

Tan, I.Y., Demchuk, A.M., Hopyan, J., Zhang, L., Gladstone, D., Wong, K., Martin, M., Symons, S.P., Fox, A.J. & Aviv, R.I. (2009) CT angiography clot burden score and collateral score: correlation with clinical and radiologic outcomes in acute middle cerebral artery infarct. *AJNR Am J Neuroradiol*, **30**, 525-531.

Zaidat, O.O., Yoo, A.J., Khatri, P., Tomsick, T.A., von Kummer, R., Saver, J.L., Marks, M.P., Prabhakaran, S., Kallmes, D.F., Fitzsimmons, B.F., Mocco, J., Wardlaw, J.M., Barnwell, S.L., Jovin, T.G., Linfante, I., Siddiqui, A.H., Alexander, M.J., Hirsch, J.A., Wintermark, M., Albers, G., Woo, H.H., Heck, D.V., Lev, M., Aviv, R., Hacke, W., Warach, S., Broderick, J., Derdeyn, C.P., Furlan, A., Nogueira, R.G., Yavagal, D.R., Goyal, M.,

Demchuk, A.M., Bendszus, M. & Liebeskind, D.S. (2013) Recommendations on angiographic revascularization grading standards for acute ischemic stroke: a consensus statement. *Stroke*, **44**, 2650-2663.
